# Post-Carnegie II curricular reform: a north American survey of emerging trends & challenges

**DOI:** 10.1186/s12909-019-1680-1

**Published:** 2019-07-12

**Authors:** Arnyce R. Pock, Steven J. Durning, William R. Gilliland, Louis N. Pangaro

**Affiliations:** Uniformed Services University of the Health Sciences, 4301 Jones Bridge Road, Bethesda, MD 20814 USA

**Keywords:** Curriculum reform, curricular revision, Undergraduate medical education

## Abstract

**Background:**

In 2010, coincident with the 100th anniversary of Flexner’s sentinel report, the Carnegie Foundation published an updated review of North American medical education and challenged medical schools to initiate further educational reforms. Specific recommendations pertained to a) ensuring standardized outcomes while allowing for individualized processes, b) integrating foundational knowledge with clinical experience, c) cultivating habits of inquiry and innovation and d) professional identity formation. As we approach the 10-year anniversary of this latest report, we sought to determine what type of curricular revisions have been emerging within the past decade and what types of challenges have been encountered along the way?

**Methods:**

In 2018, an electronic survey was sent to all 166 Liaison Committee on Medical Education (LCME) accredited North American Medical Schools, using the points of contact (educational deans) that were listed in a publicly available, Association of American Medical Colleges database. Free text comments were grouped into themes using the constant-comparative technique.

**Results:**

Sixty unique responses yielding a 36.14% response rate. The distribution of responses was proportionally representative of the distribution of public vs. private, old vs. new vs. established North American medical schools. Self-reported curricular changes aggregated into five main themes: Changes in curricular structure/organization, changes in curricular content, changes in curricular delivery, changes in assessment, and changes involving increased use of technology/informatics. Challenges were predominantly focused on overcoming faculty resistance, faculty development, securing adequate resourcing, change management, and competition for limited amounts of curricular time.

**Conclusions:**

Changes in curricular organization, content, delivery, assessment and the use of technology reflect reforms that are broad and deep. Empowering faculty to “let go” of familiar constructs/processes requires strong leadership, particularly when initiating particularly disruptive curricular changes, such as relocating the Step 1 examination or shifting to a competency-based curriculum. While North American medical schools are responding to the calls for action described in the second (2010) Carnegie Foundation report, the full vision has yet to be achieved.

**Electronic supplementary material:**

The online version of this article (10.1186/s12909-019-1680-1) contains supplementary material, which is available to authorized users.

## Background

One hundred and nine years ago Abraham Flexner published the first of two landmark documents on behalf of the Carnegie Foundation, reviewing the state of medical education in North America and Europe. The first report was released in 1910 and detailed the first-hand observations that Flexner made by visiting each of the 155 U.S. and Canadian medical schools that were in existence during the period of December 1908 and April 1910. In 2010, coincident with the 100th anniversary of this sentinel report, Cooke, O’Brien, and Irby visited a self-selected sampling of 11 U.S. medical schools and 3 academic health systems, and published their findings in the form of an updated review of North American undergraduate medical education (UME). The resulting text, titled *“Educating Physicians: A Call for Reform of Medical School and Residency*” [1], a.k.a. “Carnegie II”, focused on, and advocated, four key recommendations: 1) the need for standardized outcomes while allowing for individualized processes, 2) the importance of integrating knowledge with meaningful clinical experiences, 3) cultivating habits of inquiry and innovation, and 4) professional identity formation. These recommendations should, however, be informed by current practice to include consideration of the challenges and successes that have emerged since the publication of this report.

Since embarking on a full-fledged Flexnerian-type review of the 169 North American medical schools that are currently (as of Spring, 2019) recognized by the Liaison Committee on Medical Education (LCME) would be a challenging task, a modified approach, using an electronic survey, was utilized in the present study, to explore emerging trends and challenges associated with curricular revision in undergraduate medical education. As we approach the 10th anniversary of the release of Cooke & Irby’s review of UME, we sought to determine what type of curricular changes have been emerging within the past decade? And what challenges have been encountered along the way?

### Theoretical framework

Social cognitive theory as articulated by Torre & Durning [2] served as the theoretical framework for this work. This framework was chosen as it not only incorporated the triadic reciprocality of a) personal and cognitive, b) environmental, and c) behavioral influences on behavior, but also allows for the explicit consideration of cultural influences as well as the interactions that take place within an educational community of practice. According to Lave and Wenger [4], participants become members of such a community through legitimate peripheral participation—by engaging in meaningful activities under the (initial) guidance of more established members of the community.

From a curricular standpoint, environmental considerations leading to curricular change include the influence of new and emerging technologies, medico-legal considerations, and most importantly, changes to the frontier in which healthcare is delivered. Environmental considerations have also been noted by Bruner [3] and by Lave and Wenger [4, 5] as having cultural and collaborative elements respectively. In fact, an example of the latter involves the increasing incorporation of legitimate, early clinical participation throughout medical school curricula. Taken together, these measures can be instrumental in effecting and communicating curricular changes among and within academic institutions.

Personal factors—such as how students respond to curricular material and the associated cognitive load are also integral aspects of social cognitive theory, particularly when applied to the realm of curricular reform. Finally, behavioral influences—which not only take into consideration how students respond/react to certain teaching methodologies, but also consider that schools can learn from the collective experiences and observations shared by other schools, makes this a salient framework for this type of research.

## Methods

Consistent with best practices in survey design, a literature review (Additional file 1) was first conducted to ascertain what type of contemporary, post Carnegie II curricular revisions and challenges had already been described in the literature within the past 10 years. The resulting analysis was used to inform construction of the survey instrument. (Additional file 2).

### Survey methodology

In the Fall of 2018, an electronic survey was developed and distributed to representatives of all LCME accredited North American medical schools (*N* = 166 at the time of the survey). The survey instrument was disseminated electronically for purposes of efficiency, and to facilitate a comparable analysis, by eliciting curricular feedback at a designated point in time. The instrument sought to identify what and where some of the self-identified curricular innovations were occurring, what challenges might have been encountered, and to determine how many schools have transitioned away from a traditional, 2 × 2 (24 month predominantly basic science oriented + 24 months of primarily clinically oriented) curriculum, to a more integrated, organ-system type approach.

The survey was developed independently by the primary author (AP) and was further refined following a focused review with three colleagues representing different facets of academic medicine. One was a PhD with specific expertise in survey methodology, one was an academically oriented physician, and the third was a mid-level administrator. Additionally, two structured, face-to-face, cognitive interviews—one involving a physician colleague, and one involving a non-physician staff member, were then used to further refine the survey instrument (Additional file 2). The completed survey was subsequently reviewed by the Uniformed Services University of the Health Sciences (USU) Institutional Review Board (IRB) which determined that the protocol was IRB exempt.

The finalized survey was distributed via an electronic survey platform (Qualtrics; https://www.qualtrics.com), during the period from 6 August − 10 September 2018. The survey was sent to each of the individuals who were listed as being institutional points of contact in the Association of American Medical Colleges’ (AAMC) publicly available database (May 2018 edition) of educational deans. This database was utilized as a convenience sample and representatives of all 166 schools received copies of the noted survey. In most cases, the designated individuals were Associate Deans of Medical Education or Associate Deans for Curriculum, but in some instances the Dean of Academic Affairs or Vice Dean were listed. Approximately 5 days prior to the sending of the first survey, an introductory message was sent via email to each of the prospective respondents, alerting them to the upcoming arrival of the survey.

The surveys were subsequently disseminated at T = 0, and to non-respondents, at T = 1, 2, 3 and 4 weeks. Survey responses were tracked and acknowledged on a weekly basis, with a brief thank-you e-mail being sent to those who responded to the survey request in full or in part. In an effort to enhance the response rate, schools for which an initial response was not received at the end of the first full week received additional, modified reminder messages on a weekly basis, for up to 4 weeks. The absence of a reply after the 4th attempt was recorded as a negative reply.

The survey was comprised of forced-choice responses and free text items. Forced choice items were collated and reported in a standard manner. All of the free-text replies were initially reviewed by the primary author (AP) who conducted an independent analysis, aligning the responses into provisional themes in accordance with the constant-comparative technique. This preliminary categorization was independently reviewed and refined by a three-person subgroup (SD, LP, WG); all investigators (AP, SD, LP, WG) reviewed the final coding to ensure thematic accuracy and consensus.

## Results

The overall response rate was 36.14%, representing responses from 60 of 166 medical schools. Free text responses were reviewed and coded by the authors into three general categories: respondent demographics, curricular changes/innovations, and challenges encountered when undertaking a significant curricular revision. Within the curricular innovations and challenges categories, the authors grouped findings into themes. Thematic saturation was reached after approximately 88% of the responses were reviewed.

### Respondent demographics

Sixty-one survey responses were received, with one duplicate entry, leaving a net response of 60 participants. Using a denominator of 166, which reflected the total number of U.S. (*N* = 149) and Canadian (*N* = 17) medical schools that had been granted full (*N* = 141), preliminary (*N* = 6), or provisional (*N* = 2) accreditation by the LCME [6] by the start of calendar year 2018, the overall response rate was 36.14%.

Of the 60 unique responses, seven respondents did not include a school affiliation, inserting a “N/A” annotation in response to this question. There were also two respondents from schools in Puerto Rico and two from Canadian medical schools.

Using the US Census Bureau’s definition of geographic regions [7] survey responses were received from 9 of the 34 (26.5%) LCME accredited medical schools located in the Northeastern U.S. Twelve of 34 (35.3%) Midwestern schools replied, as did 24 of the 54 schools (44.4%) located in the Southern U.S. While 50% (2 of 4) Puerto Rican schools submitted survey responses, only 3 of the 21 (14.3%) of schools in the Western U.S. responded, as did only 2 of the 17 (11.8%) of Canadian medical schools accredited by the LCME. A summary of respondents—indicating geographical region, duration of LCME accreditation, and type of school (public vs. private) is reflected in Additional file 3 [6, 8].

When considering the representation of public versus private institutions in the survey response, survey representation was nearly equivalent with that of the U.S overall, with 62% of survey respondents emanating from public institutions and 38% from private institutions. This is consistent with national statistics [8] that indicate that 63% of the allopathic schools located within the United States are public institutions, with 37% being privately sponsored.

Finally, using the year of initial LCME accreditation as a comparison, the number of respondents from relatively new, old, or established medical schools was also found to be proportionally representative of the overall distribution within North America (Additional file 4).

### Curriculum based demographics

One of the foundational questions in the survey asked “Does your school have a standard, pre-clinical, basic science curriculum? In other words, are students required to complete a period of study focused on the basic sciences—anatomy, physiology, pathology, microbiology, immunology, etc., before starting their full-time rotations?” While the majority (83.3%) of the 54 respondents replied in the affirmative, 9 (16.6%) replied “no”. An investigative review (Additional file 5) of the pre-clinical curricular descriptions included on these schools’ websites revealed an increased emphasis on advanced forms of curricular integration, the use of longitudinal threads, early and substantive clinical immersions, and/or blended learning techniques. Moreover, several of these schools are not only integrating clinical and basic sciences in the pre-clinical curriculum but are actively incorporating elements such as health systems science along with the arts and humanities, into students’ early educational experiences as well.

**Fig. 1:**
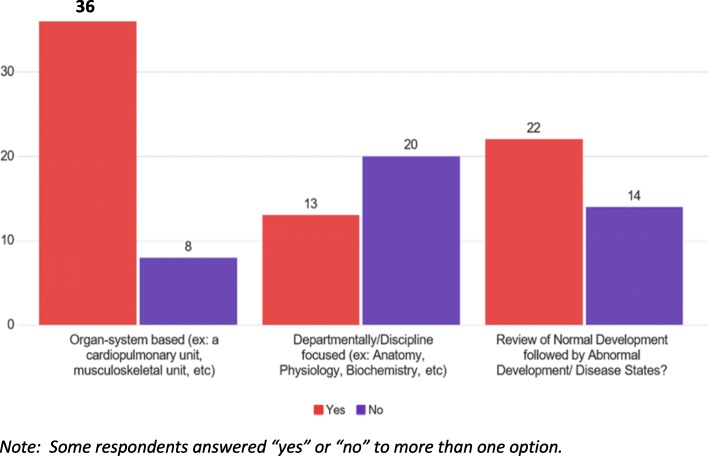
How schools with a standard pre-clerkship curriculum organize their curricular content

Of the 45 schools that indicated that they do offer a standard, basic-science oriented, pre-clerkship curriculum, the typical duration was 18 months; however one school reported use of a 13-month pre-clerkship curriculum, with 6 schools continuing to utilize a traditional 24-month pre-clerkship curriculum. The survey also sought to determine how schools tended to organize their pre-clerkship curricula—i.e. continuing to maintain a departmentally focused approach, an organ-systems type approach, or a review of normal followed by abnormal development/disease states. While some schools may be using a combination of two or more of these organizational approaches, the most commonly cited response (Fig. 1) involved use of an organ-system based approach to the pre-clerkship curriculum, with departmentally/disciplined focused presentations and/or review of normal followed by abnormal disease states comprising smaller proportions.

### Curricular changes/innovations

One of the key components of this survey involved identifying the “three most significant curricular changes” that schools had undertaken within the past 10 years, and whether they were implemented as part of an overall curricular revision. The final categorization, following the constant-comparative technique, is reflected in Table 1 and represents the consensus of all four investigators (AP, SD, LP, WG). The same approach was used in categorizing the representative examples of curricular revisions summarized in Table 2.

**Table 1:** Types of Self-Reported Curricular Changes/Innovations

Self-reported curricular changes/innovations (Themes represented by bold type)	Total No. of Responses (*N* = 122)
**Changes to Curricular Structure/Organization:**	28 (22.9%)
--Structural/Organizational Changes (*N* = 16)	
--Three Year Medical School Track (*N* = 1)	
--Shortened Pre-Clerkship Curriculum (*N* = 5)	
--Re-Alignment of USMLE Step 1 Exam (*N* = 3)	
--Increasing Opportunity for Electives in MS-3 Year (*N* = 2)	
--Resurrecting “Old” Structures/Formats (*N* = 1)	
**Changes to Curricular Content:**	37 (30.3%)
--Incorporating New or Expanded Forms of Curricular Content (*N* = 10)	
--Early Clinical Exposures (*N* = 7)	
--Establishing Longitudinal Experiences (*N* = 7)	
--Reinforcing Basic Science in the Clinical Years (*N* = 3)	
--Promoting Student Research/Scholarship (*N* = 6)	
--Emphasis on Quality and Patient Safety (*N* = 2)	
--Expanded Health & Wellness Initiatives (*N* = 2)	
**Changes to Curricular Delivery:**	41 (33.6%)
--Fostering Enhanced Curricular Integration (*N* = 19)	
--Increasing Emphasis on Active Learning/Decreased Reliance on Traditional Lectures (*N* = 14)	
--Emphasis on PBL or TBL (*N* = 6)	
--Pre-Clerkship “Boot Camp” (*N* = 2)	
**Changes to Assessment:**	13 (10.6%)
--Developing a Competency Based Assessment/Curriculum (*N* = 7)	
--Incorporating New/Altered Forms of Assessment/Assessment Tracking (*N* = 3)	
--Elimination of Traditional (Letter) Grades (*N* = 3)	
**Increasing Use of Technology & Informatics:**	3 (2.5%)
--Curriculum Mapping (*N* = 2)	
--Enhanced Use of New/Emerging Technology (*N* = 1)

**Table 2:** Examples of Recently Implemented, Self-Reported Curricular Innovations

Types of curricular change/innovation (Representative examples with participant quotes in italics; themes in bold type)	Number (*N* = 122) and percent respondents citing similar change(s)
**Fostering/Enhancing Curricular Integration**	19 (15.6%)
--Incorporating and developing distinct curricular threads (e.g. Lifestyle Medicine, Medical Decision-Making & Laboratory Medicine, Health Equity & Advocacy, Teamwork & Leadership, Healthcare Quality & Patient Safety) (Northwestern Univ)	
-- *“Alignment of histology, pathology, cell biology/biochemistry with the sequence of dissections in the anatomy course. Significant to help students integrate understanding of the inter-relatedness of these disciplines….”*	
--“*Integrated curriculum based on 90 ‘Chief Complaints and Concerns.”*	
*--“We are a new school…deliberately adopted an innovative curriculum that is highly integrated and clinical presentation-based (in systems-based units with each week’s content is derived from what a clinician would need to know, understand and apply in order to diagnose a patient with a highly relevant and motivating common clinical presentation.)” ….The instructional week is based on the Kolb learning cycle, starting with motivating context (a common clinical presentation and introductory diagnostic scheme), followed by integrated instruction in relevant new material, followed by opportunities for deliberate practice (related medical skills instruction and formative assessment), followed by application in case-based small group sessions in which students apply their new knowledge in the context of the week’s clinical presentation and diagnostic scheme."*	
**Organizational Changes** (to include shortened pre-clerkship period)	16 (13.1%)
-- Created a 4-Pillar Framework: Medical Science, Clinical Science, Health Systems Science, Health Humanities	
-- *Obliterating discipline, organ system and departmentally focused course work (and normal/abnormal organization) to form large, integrated thematic blocks that require faculty from multiple disciplines from across the basic science and clinical science spectrum to work together…."*	
--Adopting an organ-system model (vs discipline-based) approach;	
--“*C21 provides a myriad of pathways for students to choose from (3, 4, 5 years and MSTP).”*	
-- Courses based on “*themes rather than departments”*	
-- *“Creation of semester-long, very large (20 credit) interdisciplinary courses….no courses are departmentally owned…all run by the office of med ed, however the funding structure of the school has not changed, and that can be problematic.”*	
**Emphasis on Active Learning/Decreased Reliance Traditional Lectures**	14 (11.5%)
-- Asynchronous lecture delivery (UC Davis)	
-- Marked increase active learning; minimum 50% active learning throughout pre-clerkship curriculum;	
-- Use of “flipped” classroom activities	
-- Expanded use of Problem Based Learning/Case Inquiry type sessions;	
--“Lecture free curriculum” (as of July 2017)	
**Expanded Coverage of Contemporary Topics/Skills**	10 (8.2%)
-- Medical Spanish (formal instruction as part of pre-clerkship curriculum)	
-- Pain Management	
-- Palliative Care	
-- Social Determinants of Health	
-- Health Systems Science (specifically cited in 4/10 schools)	
-- Course on Public Health and Health Systems	
--Population Health (specifically cited in 4/10 schools)	
--“*Development of a primary care-population medicine program from up to 24 students each year, in which students will graduate with a medical degree as well as a Master of Science of Population Medicine—a Masters program that is currently offered nowhere else in the world. This is for students whom we expect to become national leaders in academic primary care.” (Warren Alpert School of Medicine, Brown University);*	
-- Course on Cultural Competency	
-- Course on Translational Research	
-- Professional Development course	
-- Curra Personalis Curriculum (Georgetown University School of Medicine); 1-year fellowship for up to 10 medical students; followed by opportunity to participate in longitudinal developmental activities in years 2–4. See also: https://som.georgetown.edu/CuraFellowship##	
**Enhancing/Emphasizing Early Clinical Exposures**	7 (5.7%)
-- Having students work with community agencies…means of promoting service as well as inter-professional education;	
--*“Students start seeing patients [by] week 2 of medical school.”*	
**Establishing Longitudinal Experiences**	7 (5.7%)
-- Adopting a Hybrid, Traditional Block + Longitudinal Integrated Clerkship Model (Cooper Medical School)	
-- Four Year “*integrated public health and the practice of medicine curriculum*” –includes 1- public health/practice of medicine domains, including health disparities, medical economics, occupational & environmental health, etc.)	
-- Longitudinal Primary Care Component included in Primary Care Clerkship	
-- Longitudinal Integrated clerkship for students in primary care-population medicine program	
-- Thematically organized, expanded, 12-week clerkship blocks (ex: The Medical Approach to the Patient—medicine & neurology; The Surgical Approach to the Patient—surgery & emergency medicine; Women’s & Children’s Health (OB-GYN & pediatrics); Biopsychosocial Approach to Health (primary care & psychiatry);	
--*“Longitudinal courses in ultrasound (4 years), ethics, population health, system health, medical decision making.”*	
**Promoting Student Research/Scholarship**	6 (4.9%)
-- Incorporating a Capstone Course/Research Opportunity and/or Area of Scholarly Concentration extending throughout all four years of medical school;	
--*“Introduction of 16-week mentored research experience culminated with an MD thesis for ALL students.”*	
--*Scholarly inquiry requirement for all students with 8 tracks including Design, Med Ed, Digital Health, Humanities and Healthcare systems among others." (Sidney Kimmel Medical College;* *https://www.jefferson.edu/university/skmc/programs/scholarly-inquiry/overview.html**)*	
--*“Journey(s) curriculum created space for individual student passions and faculty innovations. It uses intersessions with choice of pertinent short pertinent topical faculty driven subjects. Individual journeys periods… [allow for] pursuit [of] one of five scholarly concentrations (Health Justice, Population Health & Prevention, Medical Humanities, Medical Education Research, Business Leadership and Patient Safety). Also has room for better development ... [of]…Individual Scholarly Project.” (Georgetown University School of Medicine)*	
**Emphasis on Problem and/or Team Based Learning**	6 (4.9%)
-- Using small groups of students	
**Shortened Pre-Clinical Curriculum**	5 (4.1%)
**Eliminated Traditional (Letter) Grades**	3 (2.5%)
--Pass-Fail Pre-Clerkship Curriculum	
**Reinforcing Basic Science in Clinical Years**	3 (2.5%)
-- “Return to deep dives in Basic Science after early clinical entry” (Georgetown University School of Medicine)	
**Re-alignment of USMLE Step 1 Examination**	3 (2.5%)
**Optimized Assessments/Assessment Tracking**	3 (2.5%)
-- JustInTimeMedicine Software for dashboarding of all assessment data	
--“*Introduced an arc of high-fidelity clinical skills assessment”*	
-- “*Longitudinal progress tests of clinical reasoning”*	
**Curriculum Mapping**	2 (1.6%)
--*“Standardization of pre-clerkship curriculum to standardized examination content outline and linkage of all materials (lecture objectives, quiz and exam questions,* etc.*) to this blueprint.”*	
**Emphasis on Quality and Patient Safety**	2 (1.6%)
-- Lean Six Sigma Yellow Belt training for all M1 students (Cooper Medical School, Rowan Univ. NJ)	
**Pre-Clerkship “Boot Camp”**	2 (1.6%)
**Expanded Health & Wellness Initiatives**	2 (1.6%)
--*“We launched a health and wellness initiative integrating nutrition, exercise, and mindfulness training into our curriculum”*	
**Increasing Opportunity for Electives in MS-3 Year**	2 (1.6%)
--“*Allow students to take electives during their clerkship year…. Giving students exposure to fields they would not ordinarily have exposure to with conventional clerkships.”*	
**Three Year Medical School Track**	1 (0.8%)
-- Accelerated Competency Based Education for students interested in Primary Care (UC Davis)	
**Resurrecting “Old” Structures/Formats**	1 (0.8%)
-- Returned to stand-alone M1 Anatomy course; “*students were not mastering anatomy content” when integrated during the first two years of the curriculum;*	
**Technology Related**	1 (0.8%)
-- Issuing iPads to all students; “*curriculum is delivered to the iPads and pedagogies such as flipped scurriculum utilizing i-book, interactive videos, and team-based learning are being utilized.”*	

A range of curricular innovations was cited by survey respondents (Table 2). While all are noteworthy, the investigators believed that some of the more unique examples included the incorporation of Lean Six Sigma Yellow Belt training for all medical students (Cooper Medical School), offering students the option of completing a Master of Science in Population Health as part of a pre-selected, primary care oriented, curricular track (Warren Alpert School of Medicine), facilitating scholarly opportunities in the emerging fields of Medical Design, Health Policy, and Digital Health (Sidney Kimmel Medical College), as well as providing instruction in areas pertaining to Health Justice and Business Leadership and Patient Safety (Georgetown University).

### Challenges encountered

In contrast to the wide array of self-identified curricular revisions, responses to the question “What were the most significant challenges that you or your program encountered when contemplating any of these changes” revealed a smaller number of themes. These were reviewed, sorted, and aligned into eight over-arching categories using the constant-comparative approach with discussion and refinement by all four investigators. Their consensus is reflected in Table 3. While a large proportion of comments pertained to faculty resistance to change (“*much rested on faculty identity with their discipline-based courses”),* issues of faculty development, resourcing, and overall change management were also sources of significant challenge. Of particular note is that resistance to change was not limited to faculty, as student resistance to change (“*this is not the program I was admitted into”)* could also be a formidable factor.

**Table 3:** Significant Challenges Encountered when Contemplating Curricular Changes

Types of challenges encountered (Representative quotes in italics)	Number and percent of total respondents (*N* = 55) citing similar challenges
**Faculty Resistance to Change**	17 (30.9%)
-- *“Faculty reluctance to change. Much rested on faculty identity with their discipline-based courses.”*	
-*-“Frustration of faculty with ‘new generation learners”*	
-*-“Faculty resistance to reducing pre-clinical time”*	
-*-“…recently completed an LCME review that resulted in a perfect score. Within this context it was challenging to convince some faculty of the need for change.”*	
-*-“Getting faculty on board, fear of change, fear of loss. It took a lot of consensus building, process, and listening.”*	
-*-“The insecurity/fears of the basic science departments about losing control of courses”*	
-*-“Reluctance by anatomy faculty to move to integrated systems courses, including anatomy, rather than the stand-alone course they had for many years!”*	
-*-“Fear by basic scientists that they would be marginalized”*	
-*-“Faculty resistance to losing course control (basic science faculty) when we integrated clinical and basic science”*	
-*-“Faculty buy-in and resistance to change”*	
-*-“The biggest challenge was getting Basic Science faculty to accept the shortened science curriculum. In the first year, it seemed like they tried to sabotage the curricular change at every turn.”*	
**Faculty Development/Competing Faculty Demands/Limited Faculty Time**	9 (16.4%)
-- “*Competing other faculty demands (clinical work, research, other educational roles) that may reduce the time faculty have to develop new content or implement new teaching methods.”*	
--*“Faculty preparedness and availability continues to be the most difficult challenge to overcome. Most basic science faculty are unable or unwilling to contribute to the clinically-relevant learning experiences and clinical faculty are time-constrained, being expected to earn the clinical income that keeps the whole enterprise going.”*	
-*-“There has been insufficient attention to teacher and educational leader development”*	
**Financial Considerations/Resources**	9 (16.4%)
--*“Money—primarily compensation for clinical involvement. Clinical capacity.”*	
-*-“Getting enough time for our faculty to be small group facilitators”*	
-*-“Availability of clinical faculty for pre-clinical teaching”*	
-*-“Money!!”*	
-*-“Resources—recruiting hundreds of community-based physicians to serve as preceptors in our curriculum.”*	
**Overall Resistance to Change**	6 (10.9%)
--*“General resistance to change”*	
--*“Change is hard”*	
*--“Change management. Loss of familiar courses/structures.”*	
--*“The most significant challenge is change itself, in the eyes of students and faculty. This has been especially true as incorporated Health Systems Science into the curriculum. Students appreciate its importance in the big picture but not in the short term when Step 1 is what matters.”*	
-*-“The curriculum revision required a change in culture so that Departments no longer managed the curriculum....”*	
-*-“Reframing student and faculty expectations as we transitioned to primarily student directed small group learning. (Will the students learn enough and the right things? How will they do on national exams? Don’t they need a faculty to tell them the ‘right’ answer and exactly what they need to know?)”*	
**Technology Related**	5 (9.1%)
--*“Technological challenges—software that doesn’t interface well, glitches, or that takes some time to learn or use.”*	
-*-“Software to accomplish dashboarding and curricular organization”*	
-*-“…scheduling problems that caused much angst with the students….”*	
-*-“There has been insufficient planning for IT infrastructure needs to support the upcoming changes.”*	
**Regulatory and/or USMLE Step 1 Related Considerations**	4 (7.3%)
--*“Increased emphasis on the importance of USMLE exams…with the common result of a ‘parallel curriculum’ emerging that uses commercially available board-prep material and competes for student time.”*	
**Competition for Limited Amount of Curricular Time**	3 (5.5%)
--*“Limited time in the schedule coupled with increasing content students are expected to master”*	
*--“Compression of time/schedule in which to teach the same content.”*	
*--“Finding time for the additional curriculum. When something is added something must be cut.”*	
**Student Resistance to Change**	2 (3.6%)
*--“This is not the program I was admitted into”*	
*--“How do you keep the current students engaged and enthusiastic about their ‘old’ curriculum while trying to get faculty and staff excited about a new curriculum? The messaging is very challenging here.”*	

Despite the inherent challenges, enhancing students’ educational experience was the most common basis for implementing curricular revision, and as one respondent noted, ensuring that it “meet[s] the needs of physicians in the 21^st^ century.” Other commonly cited factors supporting curricular revision were a need to fulfill LCME related requirements, increasing student involvement in active and/or self-directed learning, addressing new or emerging curricular or societal needs, expanding opportunities for inter-professional and team-based education, and adopting new and advanced pedagogies. Or, as one respondent wrote “*our motto is ‘if it ain’t broke make it better’…so we are constantly improving our curriculum in line with advanced in adult learning and medical education.”*

## Discussion

We conducted an electronic survey to explore emerging trends and challenges associated with curricular revision in undergraduate medical education. The survey included free text responses that we analyzed using the constant-comparative technique. While our survey response rate was suboptimal, we reached thematic saturation and consensus on thematic coding to address these emerging themes and challenges. As anticipated, several of the themes aligned with social cognitive theory.

### Emerging trends

Considering all the curricular changes that responding schools have implemented, relocation of the USMLE Step 1 examination to after the core clerkships was certainly one of the more substantive and/or controversial changes, second only to the move towards competency (versus time based) progression and completion of undergraduate medical education. While administering a delayed Step 1 exam is still a relatively new phenomenon, there appears to be a small, but steady escalation of the number of schools (*N* = 18) that are now opting for this approach [9].

Another emerging trend relates to the use of competency versus time based curricular programs. One of the key advantages of shifting to a competency-based curriculum is that it allows students to progress in a more tailored and/or accelerated fashion—consistent with the 2010 Carnegie report that advocated for standardized outcomes but with individualized processes. On the other hand, doing so presumes that interim assessments predict future performance. Malone and Supri [10] also expressed concern that competency-based curricula run the risk of “teaching to the test,” and promoting a more “task based” orientation—as opposed to fostering intellectual curiosity and scientific exploration. There is also the potential for even more detailed administrative and documentary requirements—which could further diminish the amount of time faculty can devote to clinical care and in-person teaching. Competency-based education clearly has its merits, but whether it is truly reflective of the next “wave” of curricular revisions remains to be seen.

A number of schools are revising their approaches to undergraduate medical education by incorporating instruction relating to new and evolving contemporary topics (e.g. social justice, digital health, Lean Six Sigma training). There also seems to be an expanding emphasis on helping students cultivate skills associated with the business side of medicine, and on deliberately developing medical students to assume the mantle of physician leadership. The emphasis on the business of medicine is manifest by the increase of dual MD/MBA programs from six in 1993, to over 60 in 2018 [11]. The focus on leadership and entrepreneurship is exemplified by the University of Texas at Austin’s Dell Medical School which aims to cultivate a “new breed” of physician leaders/influencers. In fact, students at Dell are not only afforded a full 9 months to engage in a “Innovation, Leadership and Discovery” block, but are actively encouraged to engage in areas ranging from re-designing and innovating health care to becoming a “student entrepreneur in residence” [12]. Yet another example is that of Carle Illinois College of Medicine, a school that was specifically established to foster the development of a new cadre of physician-leaders—those who are “trained in medicine through the lens of engineering” [13], and who can actively embrace the growing interface between science, technology and medicine. Whether these modified curricula succeed in fully addressing contemporary societal needs and expectations is a question for future research.

Other notable trends include a growing emphasis on enhancing curricular integration, promoting and sustaining a life-long commitment to self-directed scholarship, creating opportunities for early, meaningful clinical exposures (a.k.a. legitimate peripheral participation) and longitudinal clinical experiences. These too, are significant, as they directly support the key tenets outlined in the most recent Carnegie report and are also consistent with social-cognitive theory.

### Identifying and overcoming challenges

Whether “letting go” involves de-constructing the familiar structure of a long-standing, course-based curriculum, or eliminating reliance on the traditionally delivered, 50-min lecture, the challenges of implementing major curricular changes are often underestimated.

In fact, one of the recurrent themes of this research involved consideration of the many obstacles that can impede the implementation of curricular reform in particular and change management in general.

When it comes to curricular revision, faculty engagement and support can be a force multiplier or a force divider. This was exemplified by the prominent reference to “faculty” when survey respondents were asked to identify the most significant challenge(s) that were encountered when contemplating curricular change. On the other hand, academic change can often be facilitated if the associated leadership team recognizes that while change is an event, transition—to a new curriculum or other major innovation, is a process. The process of implementing and managing a major transition was aptly described by William Bridges, who noted that

“The starting point for transition is not the outcome, but the ending that you will have to make to leave the old situation [curriculum] behind…[the] psychological transition depends on letting go of the old reality and the old identity you had before the change took place. Nothing so undermines organizational change as the failure to think through who will have to let go of what when the change occurs.” [14]

Social cognitive theory proved to be an apt framework for evaluating survey results. This was particularly manifest when considering the types of challenges conveyed by respondents when implementing curricular revisions, many of which reflected cultural, environmental, behavioral, and/or personal/cognitive influences. As one respondent stated, “*…the office of med ed is responsible for successful courses…[it] cannot ‘hire and fire’ faculty as departmentally ‘owned’ faculty still teach all of the courses.”* This statement further highlights the crucial under-pinning of any successful curricular revision: the need to seek, attain, and facilitate the active “buy-in” of the community of students, faculty and other key stakeholders. In fact, when it comes to change management overall, author Jim Collins perhaps said it best when he noted that:

*“Leaders of companies that go from good to great start not with ‘where’ but with ‘who.’ They start by getting the right people on the bus, the wrong people off the bus, and the right people in the right seats. And they stick with that discipline—first the people, then the direction—no matter how dire the circumstances”. *[15]

There are two key themes that readers of this paper – particularly those that might be contemplating decanal positions, might find beneficial when designing and/or modifying future medical school curricula. First, as John F. Kennedy once said, “*change is the law of life and those who look only to the past or present are certain to miss the future*.” [16] This is particularly significant for while change management can be a challenging—and daunting prospect, the potential rewards can be immeasurably positive. Second, while the more recent Carnegie Foundation report includes some salient “calls for action,” it should be noted that it concentrated on four specific processes and was based on a relatively focused survey of contemporary medical schools. So while these avenues for action should still be heeded, they should not distract nor preclude schools from continuing to innovate, and from ensuring that emerging curricula are responsive to the challenges and health needs of increasingly diverse global societies. As such, embracing opportunities to expand the boundaries of early inter-disciplinary and inter-professional curricular integration, and continuing to evaluate the efficacy of competency driven education should be viewed as pivotal foundations for future success.

The primary limitation of this study pertains to an overall response rate of 36.14% and the potential for non-response bias. This may have been partly induced by the cognitive load associated with a survey that included several self-reported, free text responses. There did, however, appear to be proportional representation with regard to the presence of full or provisional LCME accreditation, status as an “old,” “new” or “established” medical school, and status as a private versus public institution. These finding collectively suggest that these results may still be representative of other schools in the North American hemisphere. Additionally, as the data were self-reported, we may not have identified other salient trends, such as the expansion of the medical humanities in undergraduate medical education. Further, as the data sampled North American institutions, developments in medical education in other parts of the global community may not be represented and deserve further investigation.

Efforts to reduce non-response bias were undertaken in accordance with the work of Phillips et al. [17] In particular, a review of the Qualtrics captured time/date free-text entries for early versus late respondents suggested no difference in responses. Additionally, thematic saturation was achieved after approximately 88% of the responses were received.

Since it was not always apparent as to who was completing the survey, there may have also been some variability depending on individual respondents’ fund of institutional knowledge, their experience with, and/or knowledge of curricular changes at their school. Although difficult to definitively ascertain, an inherent selection bias may also have been present, as some respondents may have simply been more or less inclined to participate in the survey for personal, professional, or other reasons. It is also possible that individual responses may have been subject to an interpretive bias, as the submitted replies may have been more reflective of how respondents *perceived* curricular revisions within their respective institutions. Stated another way, what academic leaders’ *think* they are doing may not always equate to actual actions and/or effects. Use of the AAMC database may have introduced an element of bias, as it is possible that some of the respondents—the majority of whom were in academic leadership positions, may have consciously or unconsciously sought to represent their programs in the best possible manner, thereby over or under estimating the impact of salient curricular revisions and/or challenges. In this regard, the ability to visit each of the responding institutions and conduct an on-site validation—akin to the manner in which Abraham Flexner conducted his initial review, would have been helpful in ameliorating this limitation. Finally, there may be questions pertaining to the use of new or emerging pedagogies that were not addressed by the survey instrument.

## Conclusions

While the 2010 Carnegie report highlighted several key areas for educational enhancement and reform, it is evident that nearly 10 years later, definite progress has been made, but perhaps not entirely in precisely the same manner as the authors originally envisioned. For example, one of the foundational tenets of Carnegie II was a call for standardized outcomes while allowing for individualized processes. One manifestation of this call for action may in fact be the increased consideration of the merits of competency versus time-based curricula. Another may be related to the fact that many schools are now offering students opportunities to embark on specialized educational tracks (ex: rural medicine tracks, dual degree, science and engineering programs) that allow them to customize and/or accelerate their educational experience in accordance with their individual career aspirations, while still demonstrating achievement in the foundational elements of undergraduate medical education.

Carnegie II also included a call for increasing the integration of clinical and basic sciences—something that many medical schools have responded to by deliberately introducing foundational elements of clinical medicine and early legitimate (clinical) peripheral participation throughout the curriculum, starting as early as the first few days of medical school.

A third element included in the 2nd Carnegie report focused on the importance of instilling habits of inquiry and innovation. At the level of undergraduate medical education, this has been most notably associated with the deliberate inclusion of curricular time devoted to scholarly research (ex: Capstone type projects), as well as by the fact that many medical schools are now actively encouraging and empowering students to become agents of change. For some students, the focus on innovation and inquiry may be manifest by becoming medical entrepreneurs, while others may become leaders in domains specifically focused on leading social change and alleviating issues of health disparities and social justice.

Carnegie II also emphasized the importance of professional identity formation. While attaining the skills, knowledge, and attributes that have been foundational to medicine remain critically important, this study of contemporary curricular revision has also highlighted the emergence of an expanded view of professional identity formation. In other words, it is no longer sufficient for students to simply graduate medical school as competent physicians—increasingly they must also graduate with an understanding that their identity as physicians must also include an appreciation of the roles and responsibilities they will be asked to assume as leaders, innovators, and future policy makers.

In summary, while the “wheels” of curricular change may be slow to move—particularly given the perceived constraints associated with fulfilling external accreditation requirements and reviews. That said, the willingness to explore alternative delivery systems and for the medical profession as a whole, to change our thinking about what is indeed the best approach for developing the next generation of physicians, are pivotal steps in changing the process of medical education for generations to come.

## Additional files

Additional file 1: Literature Review – a summarization of the pertinent literature reviewed in support of developing the associated survey [18–22]. (DOCX 25 kb)

Additional file 2: North American Survey – word document version of the Qualtrics survey referenced in the manuscript. (DOCX 15 kb)

Additional file 3: Demographics of Responding Schools – description of responding schools by geographic area, date of initial accreditation, and status as a public versus a private school. (DOCX 34 kb)

Additional file 4: Profile of Survey Responses Based on Duration of LCME Accreditation—summarization of responding schools indicating status as an old, established, relatively new, or new medical school. (DOCX 13 kb)

Additional file 5: Schools Self-Identified as Not Having a Standard Pre-Clerkship Curriculum— brief summarization of the curriculum described on the corresponding schools’ websites and the associated weblinks. (DOCX 15 kb)

## Data Availability

The complete datasets analyzed during the current study are available from the corresponding author on reasonable request, however the majority of the data is included in the published article and the supplementary files.

## Abbreviations

AAMC: Association of American Medical Colleges
IRB: Institutional Review Board
LCME: Liaison Committee on Medical Education
UME: Undergraduate medical education
USMLE: United States Medical Licensing Examination
USU: Uniformed Services University of the Health Sciences
