# Supplementary material for: Post-Carnegie II curricular reform: a north American survey of emerging trends & challenges
Source: BMC Med Educ. 2019 Jul 12;19:260. doi: 10.1186/s12909-019-1680-1 (PMC6626342; doi:10.1186/s12909-019-1680-1)
Supplement: Supplementary file 1 — Literature Review – a summarization of the pertinent literature reviewed in support of developing the associated survey [18–22]. (DOCX 25 kb) [file 12909_2019_1680_MOESM1_ESM.docx]

**Additional File 1: Literature Review**

In order to determine whether the aforementioned questions had already been answered, a literature review was conducted. The review focused on English language publications released between 2007-2017, that were indexed in either the PubMed, Embase, Scopus, or ERIC databases. The literature review was conducted with the assistance of one of the senior USU librarians using various combinations of phrases pertaining to Undergraduate Medical Education, Medical School, Curriculum, Education, Models, Revision or Reform. The search initially yielded 1120 records, of which 770 were found to be non-duplicative. The abstracts for each of these records were sorted and reviewed, first eliminating papers that did not specifically pertain to undergraduate medical education, and then by eliminating manuscripts that did not address topics directly pertaining to contemporary curricular reform or revision. This led to the identification of a core of 64 articles that were reviewed in greater detail (Figure 1).

**Figure 1: Identification and Selection of Articles for Review**

Initial Search: 770 non-duplicative, potentially relevant citations

465 articles excluded due to focus on GME and/or CME versus UME

241 articles excluded due to focus on specific topics or courses (ex: radiology, anatomic dissection, etc), on the use of learning objectives, or due to a focus on specific instructional methods as opposed to a broader focus on UME curriculum

Abstracts of 305 articles reviewed

64 articles included in final analysis

While many of the analyzed articles commented on the cardinal aims of the 2010 Carnegie Report, none provided a cross sectional overview of contemporary curricular innovations or revisions—specifically focusing on those that might have been developed in the years leading up to or following the release of this 2^nd^ Carnegie report.

For example, Whitehead, et al^18^ provided a detailed review of some of the enduring challenges facing medical education (e.g. the need for generalists vs. specialists, challenges of an over-crowded curriculum, the burgeoning increase in biomedical knowledge, and the need for a greater emphasis on the social sciences), that have persisted over the past century. Others, such as DiLullo et al^19^, addressed the role of technology and the creation of “personal learning environments,” that can make teaching millennial students—i.e., those born between 1982-2001, more effective and rewarding. Jason & Douglas ^20^ described emerging efforts to create an International New School of Medicine (iNSoMed) that would serve as a ‘test bed’ for new initiatives and innovative strategies, although at the time of publication (February, 2015), the efforts were just commencing.

Other articles reinforced the need for advanced integration of clinical and basic sciences, highlighting, for example, the “New Integrated Curriculum,” which was implemented at Harvard in 2006 ^21^. Malone & Supri ^10^ addressed the advantages/disadvantages of a competency-based curriculum, while others such as Wackett et al^22^ highlighted the need for individual learning plans and a more intensified preparation for transition to internship and beyond. Others focused on specific revisions, describing, for example, the benefits of Longitudinal Integrated Clerkships, “flipped” classrooms, and the progressive shift from traditional lectures to small group teaching and problem-based learning (PBL). That said, even these innovations seem to fall somewhat short when it comes to fully envisioning the sweeping reform of UME proposed by Cooke, O’Brien and Irby.
