# Supplementary material for: Post-Carnegie II curricular reform: a north American survey of emerging trends & challenges
Source: BMC Med Educ. 2019 Jul 12;19:260. doi: 10.1186/s12909-019-1680-1 (PMC6626342; doi:10.1186/s12909-019-1680-1)
Supplement: Supplementary file 2 — North American Survey – word document version of the Qualtrics survey referenced in the manuscript. (DOCX 15 kb) [file 12909_2019_1680_MOESM2_ESM.docx]

**Additional File 2: Survey for U.S & Canadian Medical Schools (Microsoft Word Version)**

1. What is the name & location of your medical school? ___________________

2. Do you have a dedicated, pre-clinical, basic science curriculum? (e.g. Are students required to complete a period of study focused on the basic sciences—anatomy, physiology, pathology, microbiology, immunology, etc. prior to doing their full time, clinical rotations?)

- - Yes (proceed to next question)
  - No (skip to question 5)

3. What is the duration, in months, of your standard, pre-clinical (basic science) curriculum? ___ months

4. How is your pre-clinical curriculum organized? Please check all that apply.

- Organ-system based (ex: a cardiopulmonary unit, musculoskeletal unit, etc.)
- Departmentally/Discipline focused (ex: Anatomy, Physiology, Biochemistry, etc.)
- Review of Normal Development followed by Abnormal Development/Disease States?
- Other (please specify) _______________________

5. What are the three (3) most significant curricular changes that your medical school has undertaken within the past 10 years? Please explain why each of these changes were significant.

____________________________________________________________________________________________________________________________________________________________

6. Were any of these changes included as part of a major curricular revision/restructuring? If yes, when were they implemented? ____________________________________________________________________________________________________________________________________________________________

7. What were the most significant challenges, if any, that you or your program encountered when contemplating any of these changes? ____________________________________________________________________________________________________________________________________________________________

8. Does your current or revised curriculum have a name? If yes, what is it, and why did you choose it?

____________________________________________________________________________________________________________________________________________________________

9. What are the top three (3) curricular innovations or educational approaches that your school is most proud of? And why? ____________________________________________________________________________________________________________________________________________________________

10. Does your curriculum include any of the following? Please check all that apply:

- A Longitudinal Integrated Curriculum?
- Opportunities for Early (within the first 12 months of medical school) Clinical Participation?
- Reflective writing?
- Leadership training?
- Inter-Professional Education?
- Team based learning?
- Opportunities for students to participate in Quality Improvement/Patient Safety related projects?
- Opportunities for students to participate in a Clinical or Bench research-based project of their choosing?
- Opportunities for individual students to advance in their training, ahead of their peers, if they are able to demonstrate that they have already attained a specific skill or knowledge level?

Please explain why your school chose to implement the items that were checked: ____________________________________________________________________________________________________________________________________________________________

11. In what year did your school admit its first Class of medical students? _________

12. Do you have a picture or chart that describes what your curriculum looks like?

If so, could you email us a copy—or provide a link to the corresponding website?

{Contact information inserted here}

13. Is there anything else that you would like to tell us about your school or your medical school curriculum? ____________________________________________________________________________________________________________________________________________________________

14. May we contact you for a brief, follow-up, phone interview to discuss some of your answers?

- 1. Yes
     1. Daytime phone #: ______________________
     2. Email: ________________________________
  2. No thanks.
