# Supplementary material for: Post-Carnegie II curricular reform: a north American survey of emerging trends & challenges
Source: BMC Med Educ. 2019 Jul 12;19:260. doi: 10.1186/s12909-019-1680-1 (PMC6626342; doi:10.1186/s12909-019-1680-1)
Supplement: Supplementary file 3 — Demographics of Responding Schools – description of responding schools by geographic area, date of initial accreditation, and status as a public versus a private school. (DOCX 34 kb) [file 12909_2019_1680_MOESM3_ESM.docx]

**Additional File 3: Demographics of Responding Schools**

Note: Geographic regions pertain to those defined by the U.S Census Bureau

| **Northeastern Respondents**  **(N=9 of 34 LCME Accredited Schools in Region)** | **Date of Initial LCME Accreditation^[[1]](#footnote-1)^** | **Type of School** |
| --- | --- | --- |
| Cooper Medical School of Rowan University, NJ | 2011 | Public |
| Albert Einstein College of Medicine, NY | 1955 | Private |
| Penn State College of Medicine, PA | 1967 | State-Related |
| Lewis Katz School of Medicine at Temple University, PA | On or prior to 1942 | State-Related |
| Columbia University Vagelos College of Physicians and Surgeons, NY | On or prior to 1942 | Private |
| New York University School of Medicine, NY | On or prior to 1942 | Private |
| Yale School of Medicine, CT | On or prior to 1942 | Private |
| Sidney Kimmel Medical College at Thomas Jefferson University, PA | On or prior to 1942 | Private |
| Warren Alpert Medical School of Brown University, RI | 1963 | Private |

| **Midwestern Respondents**  **(N=12 of 34 LCME Accredited Schools in Region)** | **Date of Initial LCME Accreditation** | **Type of School** |
| --- | --- | --- |
| Northwestern University Feinberg School of Medicine, IL | On or prior to 1942 | Private |
| University of Wisconsin School of Medicine and Public Health, WI | On or prior to 1942 | Public |
| Oakland University William Beaumont School of Medicine, MI | 2010 | Public |
| Michigan State University College of Human Medicine, MI | 1964 | Public |
| University of Missouri-Columbia School of Medicine, MO | On or prior to 1942 | Public |
| Case Western Reserve University School of Medicine, OH | On or prior to 1942 | Private |
| Southern Illinois University School of Medicine, IL | 1972 | Public |
| Chicago Medical School at Rosalind Franklin University of Medicine and Science, IL | On or prior to 1942 | Private |
| Central Michigan University College of Medicine, MI | 2012 | Public |
| Boonshoft School of Medicine Wright State University, OH | 1976 | Public |
| Washington University in St. Louis School of Medicine, MO | On or prior to 1942 | Private |
| University of Kansas School of Medicine, KS | On or prior to 1942 | Public |

| **Southern Respondents**  **(N=24 of 54 LCME Accredited Schools in Region)** | **Date of Initial LCME Accreditation** | **Type of School** |
| --- | --- | --- |
| Florida State University College of Medicine, FL | 2002 | Public |
| George Washington University, DC | On or prior to 1942 | Private |
| Texas Tech University Health Sciences Center School of Medicine, TX | 1971 | Public |
| Morehouse School of Medicine, GA | 1978 | Private |
| University of Tennessee Health Science Center College of Medicine, TN | On or prior to 1942 | Public |
| Marshall University Joan C. Edwards School of Medicine, WV | 1977 | Public |
| Baylor College of Medicine, TX | On or prior to 1942 | Private |
| University of Texas at Austin, Dell Medical School, TX  (provisional accreditation, 2015) | 2015 | Public |
| East Tennessee State James H. Quillen College of Medicine, TN | 1978 | Public |
| University of South Alabama College of Medicine, AL | 1972 | Public |
| The University of Texas Health Science Center at San Antonio Joe R. and Teresa Lozano Long School of Medicine, TX | 1968 | Public |
| University of South Carolina School of Medicine, Columbia, SC | 1976 | Public |
| Howard University College of Medicine, DC | On or prior to 1942 | Private |
| University of Florida College of Medicine, FL | 1958 | Public |
| University of Kentucky College of Medicine, KY | 1960 | Public |
| University of Louisville, School of Medicine, KY | On or prior to 1942 | Public |
| University of Mississippi School of Medicine, MS | On or prior to 1942 | Public |
| The University of Texas Southwestern Medical School, TX | 1943 | Public |
| Virginia Tech Carilion School of Medicine, VA | 2009 | Private |
| Wake Forest School of Medicine of Wake Forest Baptist Medical Center, NC | On or prior to 1942 | Private |
| Charles E. Schmidt College of Medicine at Florida Atlantic University, FL | 2011 | Public |
| Georgetown University School of Medicine, DC | 1949 | Private |
| Paul L. Foster School of Medicine Texas Tech University Health Sciences Center, TX | 2008 | Public |
| Virginia Commonwealth University School of Medicine, VA | On or prior to 1942 | Public |

| **Western Respondents**  **(N=3 of 21 LCME Accredited Schools in Region)** | **Date of Initial LCME Accreditation** | **Type of School** |
| --- | --- | --- |
| University of California, Davis School of Medicine, CA | 1967 | Public |
| Oregon Health & Science University School of Medicine, OR | On or prior to 1942 | Public |
| California Northstate University College of Medicine, CA (preliminary accreditation, 2015) | 2015 | Private |

| **Puerto Rican Respondents**  **(N=2 of 4 LCME Accredited Schools in this Commonwealth)** | **Date of Initial LCME Accreditation** | **Type of School** |
| --- | --- | --- |
| University of Puerto Rico School of Medicine, PR | 1954 | Public |
| San Juan Bautista School of Medicine | 2007 | Private |

| **Canadian Respondents**  **(N=2 of 17 LCME Accredited Schools)** | **Date of Initial LCME Accreditation** |
| --- | --- |
| University of Alberta Faculty of Medicine and Dentistry, Canada | 1936 |
| University of Toronto Faculty of Medicine | 1935 |

1. The LCME was established in 1942. The first accreditation was awarded in 1943. [↑](#footnote-ref-1)
