# Supplementary material for: Post-Carnegie II curricular reform: a north American survey of emerging trends & challenges
Source: BMC Med Educ. 2019 Jul 12;19:260. doi: 10.1186/s12909-019-1680-1 (PMC6626342; doi:10.1186/s12909-019-1680-1)
Supplement: Supplementary file 4 — Profile of Survey Responses Based on Duration of LCME Accreditation—summarization of responding schools indicating status as an old, established, relatively new, or new medical school. (DOCX 13 kb) [file 12909_2019_1680_MOESM4_ESM.docx]

**Additional File 4: Profile of Survey Responses Based on Duration of LCME Accreditation**

| **Time Frame of Initial, Full LCME Accreditation** | **Number of Identifiable Respondents (N=52)** | **Percent of Total Survey Responses with Full LCME Accreditation (N=50)** | **Number and Percent of North American Schools with Full LCME Accreditation (N=158)** |
| --- | --- | --- | --- |
| On or Prior to 1942  “Old Schools” | 23 | 46% | (83) 52.6% |
| 1943-1973  “Established Schools” | 14 | 28% | (44) 27.8% |
| 1974-2004  “Relatively New Schools” | 6 | 12% | (15) 9.5% |
| 2005+  “New Schools” | 7* | 14% | (16) 10.1% |

*Excludes 2 new schools who responded, but that are currently holding provisional or preliminary LCME accreditation
