# Supplementary material for: Post-Carnegie II curricular reform: a north American survey of emerging trends & challenges
Source: BMC Med Educ. 2019 Jul 12;19:260. doi: 10.1186/s12909-019-1680-1 (PMC6626342; doi:10.1186/s12909-019-1680-1)
Supplement: Supplementary file 5 — Schools Self-Identified as Not Having a Standard Pre-Clerkship Curriculum— brief summarization of the curriculum described on the corresponding schools’ websites and the associated weblinks. (DOCX 15 kb) [file 12909_2019_1680_MOESM5_ESM.docx]

**Additional File 5: Schools Self-Identified as Not Having a Standard Pre-Clerkship Curriculum**

| Medical School | Summarization of Web-Based Description of their Pre-Clinical Curriculum | Corresponding Web Page & Curricular Name (if applicable) |
| --- | --- | --- |
| Virginia Commonwealth University School of Medicine | First year described as being devoted to “Scientific Foundations of Medicine,” focusing on structure, function, and pathophysiology of differing organ systems. | <https://medschool.vcu.edu/education/md-program/> (C^3^ Curriculum) |
| University of Kentucky School of Medicine | First 19 months focus on foundational principles of basic sciences along with a ongoing (parallel) type introduction to basic diagnosis and management strategies. | <http://meded.med.uky.edu/curriculum-overview> (Kentucky Integrated Curriculum) |
| Sidney Kimmel Medical College | 21 month, Foundations of Medicine program; integrating basic, clinical and health systems science concepts in a series of organ-system based units, each of which are 3-14 weeks in duration. | <https://www.jefferson.edu/university/skmc/md-curriculum/courses/Foundations-of-Medicine.html> (JeffMD Curriculum) |
| Southern Illinois School of Medicine | Emphasis on small groups using case-based learning, with approximately 1/3 of first year spent in clinical environments; MS-II year continues with a series of “multidisciplinary rotations of case-based, small group learning units that emphasize the basic sciences in a clinical context.” | http://www.siumed.edu/oec/about-office-education-and-curriculum-oec.html-0 |
| Michigan State University | Early & middle clinical immersions followed by core/foundational/specialized intersessions; key aspect is that curriculum follows clinical experience. | <https://curriculum.chm.msu.edu/curricular-content/overview>  (Shared Discovery Curriculum) |
| Dell Medical School | 12-month (Essentials) program includes integrated, longitudinal threads focusing on basic sciences, inter-professional education, leadership & clinical skills. Emphasis on case and problem-based learning, using blend of large and small groups. | <https://dellmed.utexas.edu/education/academics/undergraduate-medical-education/leading-edge-curriculum> (Leading EDGE Curriculum) |
| Oakland University William Beaumont School of Medicine | Organ-system oriented preclinical curriculum integrated with longitudinal instruction focusing on “the art and practice of medicine, medical humanities and clinical bioethics, the promotion and maintenance of health and personal and professional development.” | https://www.oakland.edu/medicine/curriculum/about/ |
| Northwestern University Feinberg School of Medicine | Four key elements (Science in Medicine, Clinical Medicine, Professional Development, and Health & Society) integrated throughout all three phases of the four-year curriculum. | https://www.feinberg.northwestern.edu/md-education/curriculum/index.html |
| University of South Alabama | Described as a competency-based curriculum, with first two years utilizing an organ-systems type approach with integration of clinical medicine throughout. | https://www.southalabama.edu/colleges/com/com-bulletin/curriculum-description.html |
